# Capillary-Driven Flow Microfluidics Combined with Smartphone Detection: An Emerging Tool for Point-of-Care Diagnostics

**DOI:** 10.3390/diagnostics10080509

**Published:** 2020-07-22

**Authors:** Sammer-ul Hassan, Aamira Tariq, Zobia Noreen, Ahmed Donia, Syed Z. J. Zaidi, Habib Bokhari, Xunli Zhang

**Affiliations:** 1 Bioengineering Research Group, Faculty of Engineering and Physical Sciences, University of Southampton, Southampton SO17 1BJ, UK; 2 Institute for Life Sciences, University of Southampton, Southampton SO17 1BJ, UK; 3 Department of Biosciences, Comsats University Islamabad Campus, Islamabad, Pakistan; aamira_tariq@comsats.edu.pk (A.T.); zobia_noreen@comsats.edu.pk (Z.N.); ahmeddonia123@yahoo.com (A.D.); habib@comsats.edu.pk (H.B.); 4 Institute of Chemical Engineering and Technology, University of the Punjab, Lahore, Pakistan; zohaib.icet@pu.edu.pk

**Keywords:** microfluidics, point-of-care diagnostics, antimicrobial resistance, lab-on-a-chip, capillary-driven flow, capillary action, detections, smartphone imaging

## Abstract

Point-of-care (POC) or near-patient testing allows clinicians to accurately achieve real-time diagnostic results performed at or near to the patient site. The outlook of POC devices is to provide quicker analyses that can lead to well-informed clinical decisions and hence improve the health of patients at the point-of-need. Microfluidics plays an important role in the development of POC devices. However, requirements of handling expertise, pumping systems and complex fluidic controls make the technology unaffordable to the current healthcare systems in the world. In recent years, capillary-driven flow microfluidics has emerged as an attractive microfluidic-based technology to overcome these limitations by offering robust, cost-effective and simple-to-operate devices. The internal wall of the microchannels can be pre-coated with reagents, and by merely dipping the device into the patient sample, the sample can be loaded into the microchannel driven by capillary forces and can be detected via handheld or smartphone-based detectors. The capabilities of capillary-driven flow devices have not been fully exploited in developing POC diagnostics, especially for antimicrobial resistance studies in clinical settings. The purpose of this review is to open up this field of microfluidics to the ever-expanding microfluidic-based scientific community.

## 1. Introduction

Point-of-care (POC) diagnostics enable rapid diagnosis and monitoring of the patient, e.g., at the clinic, in the field or even at home and provides on-site results to the operator instantly. POC diagnostics must provide quick and simple-to-operate testing without the requirements of laboratory technicians or sophisticated instruments [1]. Typical examples of POC devices include glucose monitoring devices and lateral flow test strips, which are widely available in the market [2]. The POC market is expanding worldwide and is predicted to be worth USD 46.7 billion in 2024 [3]. As their key features, POC devices must be portable, simple to fabricate and operate [4,5,6], accurate and provide results within minutes or hours, leading to the well-informed clinical decisions. These allow earlier knowledgeable treatments providing better chances to improve the health of patients as compared to the less ill-informed procedures performed while waiting for the clinical laboratory results [7]. One of the critical examples of this type of treatments is the use of broad-spectrum antibiotics for the treatment of bacterial infections where the results can take a few days to weeks to come from central clinical laboratories [8]. 

Microfluidics refers to the study of fluidics in micrometre channel dimensions and has been significantly developed for the miniaturization of chemical and biochemical assays offering robust, cost-effective and sensitive assays [9]. In general, these systems have been developed to provide high-throughput, parallel, and automated chemical analyses. Microfluidics has played a crucial role in the development of POC diagnostics by offering smaller sample volume consumptions and combining multiple sample-processing steps into a single device [10]. The commercialization of microfluidic devices has been attempted for various quantification platforms, including tests for numerous small molecules, protein biomarkers, nucleic acids and even cells. However, the commercialization of microfluidic-based POC devices has not been entirely successful, largely due to the requirement of handling expertise and complex miniaturized fluidic management systems [11]. 

Capillary-driven flow microfluidics, on the other hand, is a type of microfluidics which works on the principle of capillary action that allows the movement of fluids in capillaries or microchannels without the requirement of external pumping mechanisms [12]. This type of microfluidics offers the possibility of pre-coating of reagents into the microchannels, bringing forward ready-to-use devices for POC diagnostics ever promised by the scientific community for decades. The ideal type of capillary-driven flow-based device for POC diagnostics must provide results within a few hours or even minutes while the patient remains in the clinic, and on-spot diagnostic decisions can be made almost instantly. This review is mainly focused on the capillary-driven flow dynamics and current emerging devices for POC diagnostics, especially in tackling antimicrobial resistance studies via portable/smartphone-based devices. 

## 2. Principles of Capillary-Driven Flow Microfluidics 

Capillary-driven flow microfluidics works on the principle of capillary action that allows the movement of fluids in capillaries/microchannels without the requirement of pumps/external pressure. The liquid flows into the capillaries due to the surface tension and the wetting properties of the capillary, which overcome the effect of gravity and viscosity of the liquid. The factors which affect the liquid rise in capillary include the surface properties of the capillary, the types of fluid and the internal geometry of the capillary. Capillary-driven flow is continuous when initiated and stops at the filling of the capillary without the requirement of external control, generating a fully autonomous liquid handling system [13].

**Figure 1:**
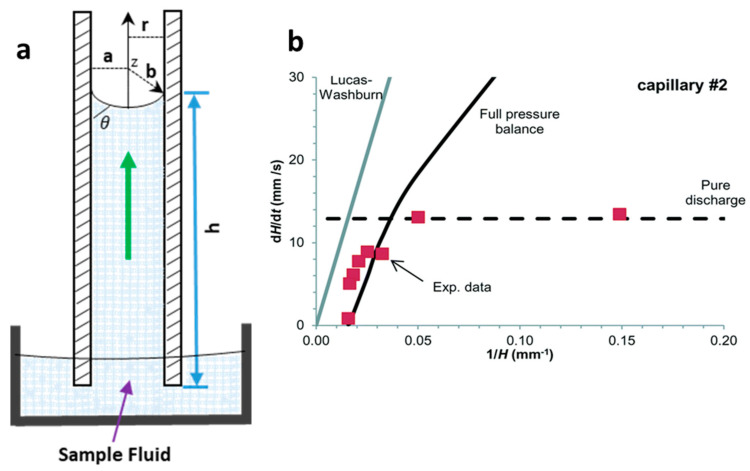
Capillary-driven flow dynamics. (**a**) Schematic of capillary-driven flow principle illustrating liquid rise in a capillary, height of the liquid rise (h), meniscus and contact angle at the liquid−air interface (θ) and the radius of the capillary (r); (**b**) Superficial flow velocity (dH/dt) modelling using Equation (3) predictions at full pressure balance in the capillary. The Lucas−Washburn equation is unable to predict the liquid rise in the capillary due to the absence of gravitational effect in the equation. Reproduced with permission from [16].

Initially, the kinetics of liquid flow along capillary were mathematically determined by the Lucas−Washburn equation, relating the capillary pressure with the fluid viscosity pressure drop at the meniscus [14]. Here, capillary pressure is the primary source of the liquid rise in the channels, which is generated by the difference in energy of liquid and air surfaces in a capillary. The most common law for a capillary rise is known as Jurin’s law [15], which relates the height of the capillary with the surface energy at the interface of the two fluids (liquid and air), the contact angle at the meniscus between two phases, capillary radius, density and gravitational acceleration on the capillary (Figure 1a), as expressed in Equation (1). The Young−Laplace equation is a widely used mathematical model (Equation (2)) that relates the capillary pressure with an internal radius of the capillary, surface tension and the dynamic contact angle at the meniscus of the liquid [16].

(1) $$h=\frac{2\gamma \cos\theta }{\rho gr}$$

(2) $$h=\frac{\gamma \cos\theta }{\rho g}\left(\frac{1}{a}+\frac{1}{b}\right)$$

where h is the height of the liquid rise; γ is the surface tension between the liquid and the air interface; θ is the contact angle at the interface; r is the radius of the capillary; g is the gravitational acceleration in the capillary; ρ is the density of the rising liquid; a and b are the width and depth of the capillary or microchannels, respectively (Figure 1a).

The Laplace pressure drop (ΔP_L_) is a combination of pressure rise in the capillary and pressure drop due to the adhesiveness/friction of the surface. Reis et al. [16] modelled an equation using fluoropolymer-based capillaries to measure the superficial flow velocity (u) based on the Young−Laplace equation (Equation (3)), as shown in Figure 1b. The authors also showed that the Lucas−Washburn model was unable to propose a maximum liquid rise in the capillary because of the absence of the maximum liquid height in the modelled equation.

(3) $$u\left(t\right)=\frac{d_{h}}{32\mu }\left[4\gamma cos\theta \frac{1}{h}-\rho gd_{h}\right]$$

The liquid rise in the capillaries with hydrophilic surfaces due to surface adhesiveness can be matched with the capillaries with hydrophobic surfaces, which tend to empty the capillary via gravitational forces. Figure 1b shows an example of modelling of superficial velocity based on Equation (3) for the full pressure balance. The authors predicted that the liquid rise up to 40% was driven via Laplace pressure, however, after an equilibrium Laplace height was reached, the liquid flow upward was driven by surface tension with superficial flow velocity (dH/dt) following the model Equation (3) [16].

Several materials have been used for passive pumping into microchannels, such as wool [17], cotton yarn [18], polyester [19] and paper [20]. These materials always allow fluid movement due to the hydrophilicity of the liquid sample. However, these methods provide a less stable flow rate, dead volume and the flow rates are fixed during the fabrication of these materials [21]. On the other hand, capillary-driven flow in microchannels occurs when microchannels are closed, or tubing is used, and the fluid stops upon completely filling into the microchannels. In this case, the microchannel must be significantly bigger than the width of the microchannel to initiate the flow, even if the microchannel is hydrophilic to the sample liquid. For the open microchannels, the Young−Laplace equation must be satisfied to initiate the fluid movement [22]. The flow rate in microchannels/capillaries is also not constant and is fixed during fabrication of devices but offers several benefits over materials as mentioned earlier, such as low dead volume, less contamination, and flow rate reproducibility between microchannels [22]. 

Capillary-driven flow has several limitations, including backflow, cross-contamination and asynchronous fluid movement inside microchannels. These problems are mainly caused by pressure differences at inlets and largely depend on the characteristics of the sample liquid [23]. Several strategies have been reported to overcome backflow problems, such as using a small flow bridge or vacuum storage [24,25]. Flow rate variability in microchannels also limits capillary flow control as mentioned above, and this problem can be circumvented by the integration of flow resistance mechanisms inside microchannels [26], varying microchannel dimensions [12], or the incorporation of porous materials at the outlets to absorb liquids [27]. Furthermore, stability and durability of capillary flow devices such as microchannels fabrication, reagent cross-linking, storage and stability must be considered for complex chemical/biochemical assays.

## 3. Recent Advances in Capillary-Driven Flow Microfluidics

**Figure 2:**
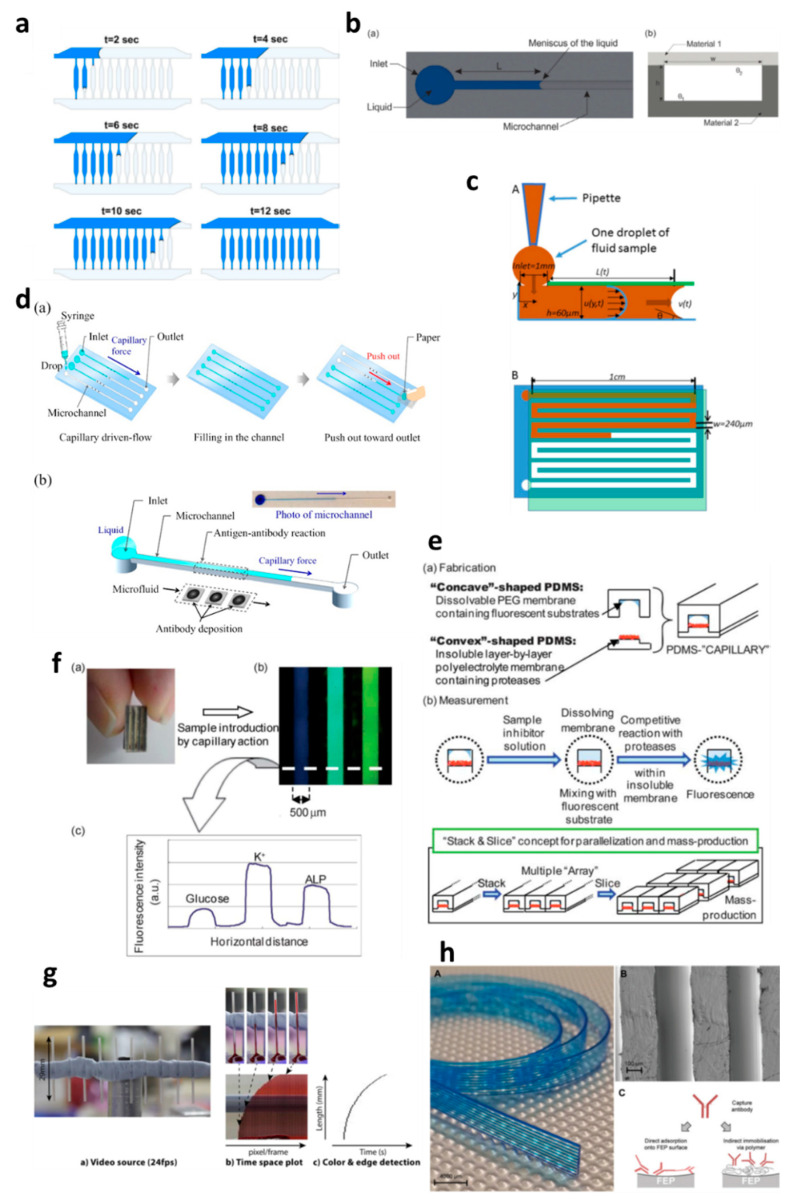
Applications of capillary-driven flow microfluidics. (**a**) computational fluid dynamics (CFD)-modelled filling of microchannels at multiple time points. Reproduced with permission from [28]; (**b**) Optical viscometer. Schematic of the microchannel with the capillary flow (**a**), illustration of the microchannel fabrication with two different substrates (**b**). Reproduced with permission from [29]; (**c**) Capillary flow pressure-driven zebrafish-blood-loaded microchannels. Side and top view of the sample pipetting and fluid flow in the microchannel (**A**,**B**). Reproduced with permission from [30]; (**d**) Illustration of the antibody immobilization process (**a**) with single-channel schematic (**b**). Reproduced with permission from [33]; (**e**) Combinable-polydimethylsiloxane (PDMS) capillary sensor array concept illustration. Reproduced with permission from [34]; (**f**) Combinable-PDMS capillary sensor array analysis of serum components via imaging (**a**) and fluorescence microscopy (b-c). Reproduced with permission from [35]; (**g**) Blood volume collection using glass capillaries. Capillary filling with EDTA blood (**a**) with the capillary filling plot of time−space (**b**) and color threshold levels and edge detection (**c**). Reproduced with permission from [41]; (**h**) Fluoropolymer microcapillaries illustration with flat ribbon blue dye-filled (**A**), field emission gun scanning electron microscopy (FEG-SEM) image (**B**), and capture antibody immobilization strategies (**C**). Reproduced with permission from [42].

Capillary-driven flow microfluidics has been developed predominantly for biomedical applications, e.g., numerical modelling of capillary-driven flow for polymerase chain reactions (PCR) inside capillaries was performed [28]. The authors modelled PCR in hydrophobic polydimethylsiloxane (PDMS) microchannels via computational fluid dynamics (CFD) which were also experimentally tested to compare results, as shown in Figure 2a. PCR mixture was introduced into the microchannels, and the liquid meniscus was recorded to fill all the channels in 12 s, which matched well with the modelled meniscus movements. In another development, capillary-driven flow devices were built to measure the viscosity of the fluid based on capillary action in microchannels [29,30]. A micromachined polymethyl methacrylate (PMMA) chip was used to create a capillary flow inside microchannels, and the meniscus was monitored via an automated optical system (Figure 2b) [29]. In this study, a small volume (26 µL) of the sample was loaded into the microchannels, and viscosity was measured by recording the fluid travel time between two fixed points. In contrast, Lee et al. [30] developed a capillary flow microfluidics device to measure zebrafish blood viscosity in microchannels (Figure 2c). The device was able to use small sample volumes (2 µL) and conduct continuous measurements to study the viscosity changes during embryonic development. Furthermore, the device was able to draw up fluids at higher shear rates, and the measurements were achieved in less than 2 min. Such optically enabled capillary flow systems have the capacity to be used as portable systems for POC diagnostics and analysis in chemical/pharmaceutical industries.

Polymethyl methacrylate was also used to fabricate a capillary flow device for nucleic acid biosensing applications having small microfluidic channels consisting of sealed reagent-loaded pads [31]. The system was incorporated with magnetic nanoparticles, which were released upon sample entry into the microchannel, and horseradish peroxidase (HRP) and hydrogen peroxide were mixed in a channel yielding potentiostat detectable species. This type of biosensor based on capillary-driven flow has the potential of miniaturization and commercialization for POC applications. Open microfluidics channels have also been developed for capillary flow [32], but they have disadvantages, such as sample evaporation, contamination and handling difficulties. Therefore, open microfluidics is not discussed further in this review, which is instead focused on capillary flow inside closed microchannels and capillaries. 

Several biomedically relevant biomarkers were studied via capillary-driven flow microfluidics [33,34,35,36,37,38]. A capillary flow immunoassay microchip was developed by Fuchiwaki et al. [33] to quantify procollagen type I C-peptide from blood samples. Laser ablation was used to immobilize the antibody on the surface of the PMMA microchannels, which allowed the reaction via movement of liquid in the channels, as shown in the schematic (Figure 2d) [33]. The liquid sample was dropped on the channel inlets which travelled towards the antibody-coated region via capillary force, incubated and flushed out via the outlet and paper absorption. Different concentrations of the peptide (0–600 ng mL^−1^) were calibrated by multiplexing the channels on a single platform and injecting them into parallel microchannels (Figure 2d). Enzyme inhibitor assays were also developed on capillary flow systems to enable single-step analysis and multiplex them for commercial purposes (Figure 2e) [34]. The device was made of polydimethylsiloxane (PDMS) and was named a “combinable PDMS capillary sensor”. In this study, fluorescent substrates were immobilized on the membrane in the microchannels and the enzyme inhibitor solution is drawn into the microchannel via capillary force. The inhibitor solution dissolves into the membrane where the reactions occur, and detection is performed in an optically clear microchannel. This method shows the potential to multiply the assays by simply cutting long PDMS microchips coated with reagents and flowing samples in parallel. A similar PDMS capillary flow device [35] was also developed for the detection of serum biomarkers, such as glucose, potassium and alkaline phosphate assay (ALP). In this device, the PDMS was mixed with a black reagent (India ink) to render the PDMS black, and the channel surface was coated with silver layers to enhance the fluorescent signals and sensitivity of the assay (Figure 2f). The sample was introduced into the microchannels via capillary flow, and the intensities of three biomarkers were attained in parallel (Figure 2f). 

Huang et al. [36] developed a capillary flow device for DNA probe detection using ordered silicon microcapillary array to control the flow of the liquid. The authors simply placed a drop of sample on the inlet (20 µL) which travelled in the microchannel with DNA probe spots (100 nL) and achieved rapid DNA detection. Recently, Soares et al. [37] developed an ultrasensitive and single-step capillary flow device for biosensing applications. The authors used bead-based technology to control the capillary flow and performed a competitive assay using only a 4.5 µL sample. The system is robust and bears a huge potential for POC diagnostics due to its turn time of 70 s per assay. 

Additionally, the capillary flow has also been used for blood plasma separation in microfluidic channels [38,39,40], such as that developed by Madadi et al. [38], to separate plasma using 5 µL of the sample, generating a plasma of 0.1 µL for diagnostic applications. The authors used microchannel-integrated micropillars to confine red blood cells and validated the separation via the detection of thyroid-stimulating hormone. Delamarche’s group [39,40] further developed plasma separation attached with immunodiagnostic devices, where C-reactive protein (CRP) was quantified by using 5 µL of human serum extracted from a blood sample and 3.6 nL of reagents solution deposited on the chip [39]. The system was able to detect low concentrations within 3 min by flowing the liquid via capillary force in the microchannels. Furthermore, a capillary flow bead-based immunoassay device for diagnostics has also been developed by the same lab [40]. These types of devices offer multiple biomedical applications with a possibility of detection via smartphones or handheld devices.

Glass capillaries, which are widely available in the market, were also utilized for capillary flow. Lapierre et al. [41] used bare glass capillaries to collect blood samples in the capillaries, as shown in Figure 2g. The authors generated profiles of blood movement in vertical capillaries, studied the effect of ageing of glass on the liquid rise and modelled the liquid rise parameters using standard equations (Equations (1)–(3)). However, fabrication of glass microchannels for POC applications is generally costly and requires tedious glass intrusions and solvent bonding. In contrast, fluoropolymer microcapillaries (FEP) have been coated with reagents to achieve hydrophilic surface inside the capillaries which can load aqueous samples rapidly (Figure 2h) [16,42,43,44,45]. Pivetal et al. [42] converted the surface of FEP microcapillaries by coating with a layer of polyvinyl alcohol (PVA) and cross-linked reagents for analyte detections (colorimetric or fluorescent), such as the detection of prostate-specific antigen (PSA) [43,44] and cytokines [45]. These FEP capillaries were injected with multiple solutions, such as PSA standard, detection antibodies, enzyme complex, washing solutions and enzymatic substrates, which were injected in all channels simultaneously. Additionally, FEP microcapillaries were placed vertically in the blood sample to draw up the liquid for ABO blood typing [16]. When the liquid rose into the capillaries, the reagents were released into the sample fluid and reacted with biomarkers to produce colour/fluorescent signal, which was detected via microscope or portable/smartphone systems. FEP microcapillaries illustrate a great potential for integration into point-of-care testing devices and offer the possibility of inexpensive biochemical analyses.

## 4. Tackling Antimicrobial Resistance in Capillary-Driven Flow Devices

Microfluidics has been successfully applied for antimicrobial resistance (AMR) studies for the development of POC diagnostic devices. Still, current devices lack the simplicity and ease of operational procedures and hence are limited to laboratory settings [46,47,48,49,50,51,52]. Antibiotic susceptibility testing (AST) is considered to be the most widely used approach to investigate the efficacy of a single antibiotic or combination of antibiotics to find the most effective treatment for bacterial infections. Broth dilution, disk diffusion, and commercially automated systems are examples of current AST techniques [46]. These techniques have been highly standardized, yet the results are obtained after a long incubation time (15~20 h). This is due to the requirement of the bacterial population to reach the minimum detectable growth level. Scientists usually depend on measuring the optical density (OD) for sensing bacterial population with a limited detection of 10^7^ colony-forming units (CFU/mL) [46,53]. Through keeping cells to a micrometer scale level, microfluidics allows individual bacterial division at early stages, which eventually lowers AST time. This could be performed within just a few hours via the indirect and direct monitoring of cell growth. Direct optical imaging is simple and can be performed on all clinical isolates. However, this is not the case in some indirect monitoring methods, due to the requisite of genetic, immunoassay modification, and complex experimental setups, meaning that some indirect methods are usually limited [46,54]. 

The short doubling time of bacterial cells reduces AST time. Thus, the direct observation of single-cell division within the entire bacterial population is of pivotal importance. Immobilization of bacteria on-chip is required for time-lapse growth observation of a single bacterium because of the high motility of several bacterial types. Single-cell trapping has been found to be feasible in microchambers, droplets, channels/tracks or traps [47,48,55,56]. Moreover, the confinement and capture of single *Escherichia coli* bacterium in microfluidic channels were performed in several studies with the help of dielectrophoresis (DEP) [57,58]. Under these conditions, *E. coli* AST was completed in 5 h [57] and 1 h [58] via observing the growth of single bacterium with the help of time-lapse microscopy. Agarose was also used to encapsulate single bacterium cells on-chip [49,59]. After gelation, culture media and antibiotics were added by perfusion via the gel and bacteria growth was observed for the determination of the minimal inhibitory concentrations (MICs) of several types of antibiotics on *Pseudomonas aeruginosa*, *Escherichia coli*, *Staphylococcus aureus*, *Klebsiella pneumoniae*, and *Enterococcus* spp. within only 4 h. However, scientists found tracking the growth of multilayer single bacterium cells in a 3D gel to be burdensome; therefore, the development of a polydimethylsiloxane (PDMS)−membrane−coverslip sandwich structure was required as a way to confine monolayer single bacterial cells [50,60,61]. The recent study integrated a gradient formation system to a similar chip assembly as a way for the determination of the minimum inhibitory concentration (MIC) and half-maximal inhibitory concentration (IC50) of amoxicillin on *E. coli* in just 4 h. PDMS microchannels, which were perfused with drug solutions (in the “source” channel) and culture medium (in the “sink” channel), were applied to set up a steady antibiotic concentration gradient in an agarose gel layer and antibiotic diffuses to the bacterial monolayer underneath. On the contrary to the determination of antibiotic resistance in terms of bacterial growth, monitoring cell death, which is achieved by the use of enzymatic and mechanical stress on a microfluidic chip, can facilitate rapid identification of antibiotic-resistant bacteria within 1 h [51]. Rapid AST is not the only advantage point of microfluidics. In fact, the MIC determined by microfluidic AST has been found to be comparable to those by conventional methods for standard Clinical Laboratory Standard Institute (CLSI) strains [49,52,62,63]. 

**Figure 3:**
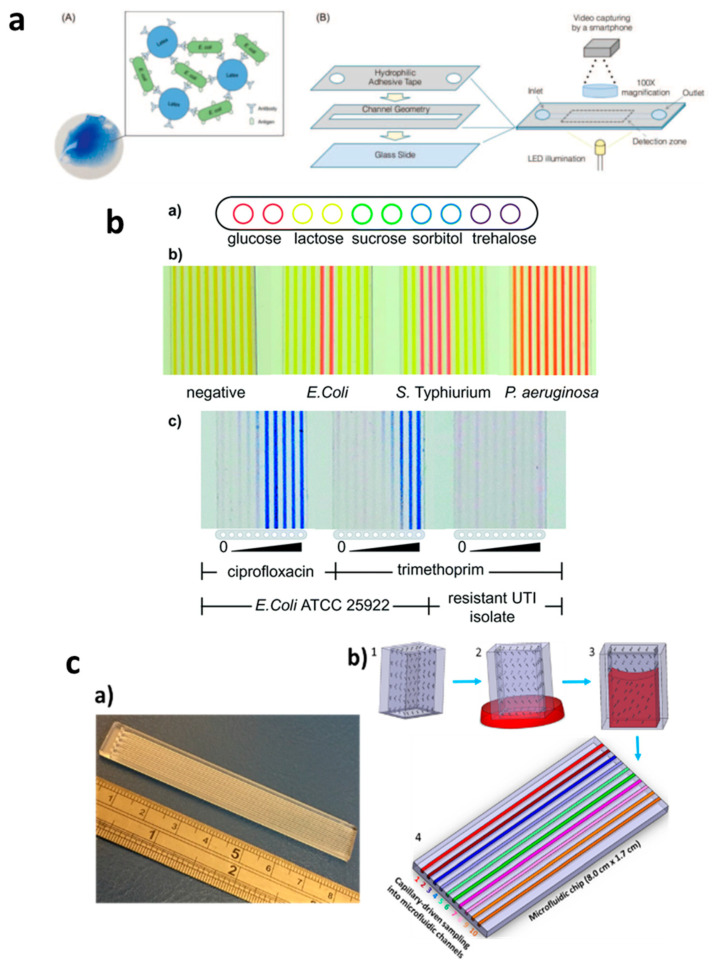
Applications of capillary-driven flow microfluidics for tackling antimicrobial resistance (AMR). (**a**) Immobilization of antibodies via latex agglutination test in which latex particles agglutinate upon the binding of the antibody with antigens loaded into the microchannel with the capillary flow (**A**,**B**). Reproduced with permission from [64]; (**b**) Fluoropolymer microcapillary-based identification of bacterial samples and antimicrobial susceptibility testing (**a**–**c**). Reproduced with permission from [16]; (**c**) Chip-and-dip capillary flow device fabrication and working schematic for beta-lactamase activity testing in microchannels (**a**,**b**). Reproduced with permission from [66].

In contrast, capillary-driven flow microfluidics has the potential to provide robust AMR devices for POC diagnostics. Several such studies were performed; Pak et al. [64], for example, developed a latex agglutinating for the quantification of *E. coli* contamination levels in samples. In this study, agglutinates were formed via antigen binding to functionalized particles (Figure 3a). The study was performed in microchannels using reagent-coated hydrophilic adhesive tape and detecting samples in a detection zone using smartphone imaging. Reis et al. [16] developed the Lab-on-a-Stick platform for antimicrobial testing via detection in an imaging scanner, as shown in Figure 3b. The authors used FEP microcapillaries coated with reagents of various types in a single microchip and successfully used it for cell-based assays by simply dipping it into a single sample and performing ABO blood typing, microbial detection and minimum inhibitory concentrations (MIC) of antibiotics. Pivetal et al. [65] also evaluated the effect of *E. coli* concentration (urine sample) on antimicrobial susceptibility testing (AST) using a novel lab-on-a-comb platform. Multiple FEP microcapillaries were used to identify AST profiles, which were found to be in comparison with standard technologies and within the clinical range of the patient urine (103–108 CFU/mL). Recently, our group developed a Chip-and-Dip device fabricated with PMMA, and the sample loading was realized by simply dipping the chip into the sample solutions (Figure 3c) [66]. By coating microchannels with nitrocefin, it enabled beta-lactamase activity to be monitored in the microchannels. We envisage that this type of capillary flow device fabricated with cheap materials can be coated with various reagents; thus, a variety of analytes can be detected in a single chip with a multichannel microchip using suitable portable/handheld detection systems [67].

## 5. Smartphone-Based Detection: A Crucial Component for Capillary-Driven POC Diagnostics

Optical microscopy has been routinely used for imaging and detection inside microfluidic devices and has been a critical component of lab-on-a-chip devices [68]. However, due to the bulk, expensive and expertise-dependent nature of the optical microscopy, its usage has been limited to central laboratories/hospitals [69,70]. Smartphone-based detection systems have recently emerged as alternative tools to these conventional microscopes, providing bright-field microscopy [71,72] as well as fluorescent imaging [73,74]. Smartphones are currently manufactured at much lower costs with built-in cameras, providing options for developing simple-to-operate mobile health devices [75]. Day by day, smartphones are incorporating additional features to allow data handling and signal processing and require minimal attachments to the phone’s camera [76]. Several smartphone-based detection systems have been developed for biomedical applications [77,78,79], especially for antimicrobial resistance studies; for example, Steve et al. [80] developed a smartphone-based system for the detection of *Klebsiella pneumoniae* in 78 patients. The authors used microtiter plates for liquid handling, and a holder was 3D printed for attachment to the smartphone and placement of a microtiter plate. The system was made of an array of light-emitting diodes (LED) for sample light illumination, and an optical fiber was used for output light capturing and detection via the smartphone camera. This technique allowed faster turbidity measurements and accurate MICs and ASTs from the microwells. In another study [81], bacterial cell growth in arrays of gas-permeable microwells was studied using a smartphone-based system. The study was performed by coating microwells with antibiotics and culturing bacteria with a substrate (colorimetric) producing a signal, which was detected by a smartphone. The authors successfully obtained profiles of ASTs for uropathogens from urinary tract infection (UTI) patients. These type of studies pave the way for the miniaturization of MIC determinations and AST profiling inside microchannels, reducing sample quantities, detection intervals and handling requirements.

**Figure 4:**
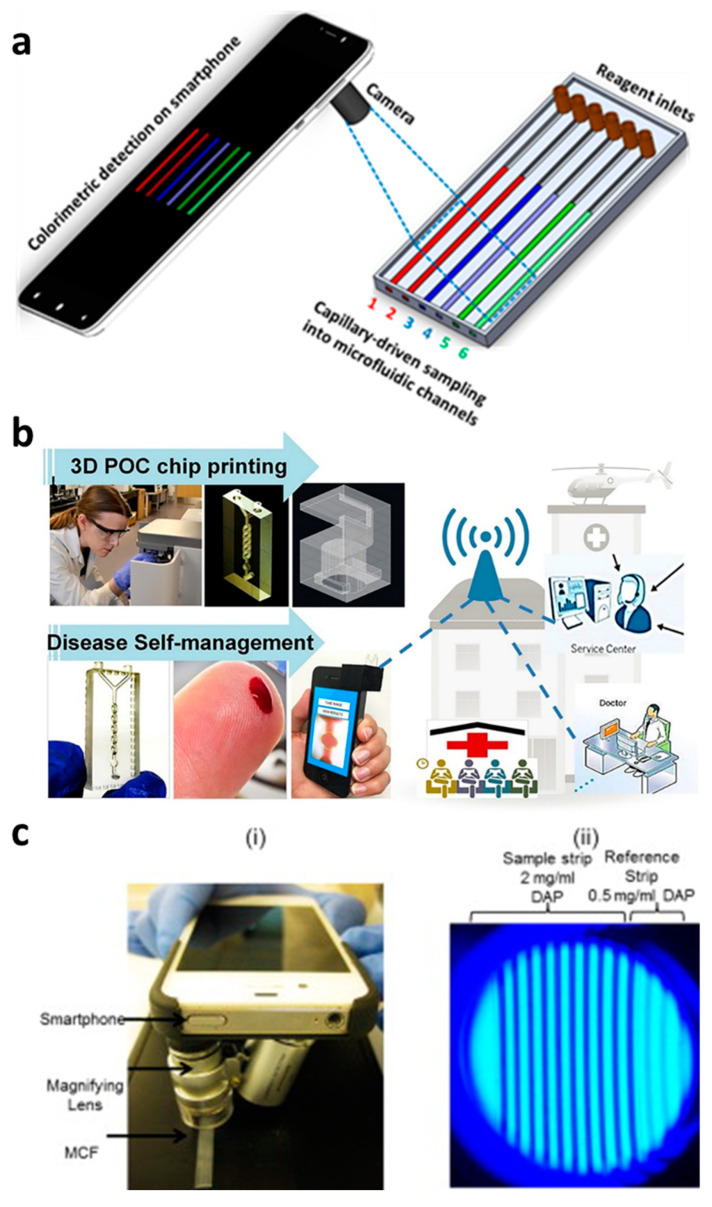
Smartphone-based capillary-driven flow microfluidics. (**a**) The transient filling of a microchannel at six different time points obtained using the CFD model; (**b**) smartphone-based detection system for anemia detection in microchannels via capillary flow. Reproduced with permission from [83]; (**c**) Smartphone-based fluoropolymer microcapillary detection system (**i**) and an image of fluoropolymer microcapillaries (FEP) filled with 2,3-diaminophenazine (**ii**). Reproduced with permission from [43].

Capillary-driven flow devices hold a great promise in developing POC diagnostics, but still rely on accessories to detect and analyze samples and hence limit the real-time applicability of these devices in clinical settings [80,81,82]. Therefore, we have proposed that smartphone-based capillary flow device, which would be an ideal option for rapid image capturing and data analysis (Figure 4a,b). Several studies using smartphone-based imaging in capillary flow devices have been performed, such as the quantitation of the prostate-specific antigen in FEP microcapillaries, beta-lactamase activity in microchannels and anemia diagnosis [43,66,83]. Plevnial et al. [83] developed a standalone POC diagnostics system via integration of a 3D-printed capillary flow device with smartphone imaging, as shown in Figure 4b. The authors demonstrated the auto-mixing of reagents with blood in a capillary flow device in a minute amount of time and performed anemia diagnosis. The blood sample (5 μL) was obtained via a finger-prick method, diluted and placed in a microwell where the blood Hgb levels were detected via oxidation-reduction reaction with 3,3′,5,5′-TMB and smartphone imaging. In another study [43], a smartphone-based detection system to quantify prostate-specific antigen via colorimetric assay reaction was developed (Figure 4c). The smartphone was also converted into a fluorescent imaging microscope and hence is suitable for the study of multiple fluorescent-based assays in capillary flow systems. These types of device designs and smartphone analysis systems can provide an ideal platform for numerous disease diagnostic systems.

## 6. Conclusions and Future Challenges

Point-of-care (POC) diagnostics allow the testing of patients in real-time, such as lateral flow assays widely used in the home or clinical settings. However, they have limitations, including the analysis of active bacterial/pathogenic cells, and hence are not applicable for fluid flow applications. Microfluidics plays a vital role in the development of miniaturized POC devices, but handling and accessory requirements make it less practical for deployment in clinical settings. Capillary-driven flow microfluidics has emerged as an alternative to these cumbersome techniques and offers robust, cost-effective and simple-to-operate microfluidic devices. This type of microfluidics has potential for adaptation towards point-of-care diagnostics, especially for tackling antimicrobial resistance, and requires global scientific attention. Such devices can be pre-coated with specific reagents and multiplexed for multiple analyte detections at minimum costs and fewer labour requirements. Furthermore, the elimination of pumping and fluid control systems makes this technology suitable for smartphone/portable detection, as the devices can be simply inserted into the optical detection systems holders attached to smartphones. 

One of the most prominent challenges for capillary-driven flow microfluidics is the stability and control of flow rates in microchannels. The flow rates largely depend on inlet pressures and are fixed during fabrication of microchannels. Several device modifications have been attempted to overcome this challenge, such as by varying the dimensions of microchannels to control fluid movement [12] and the incorporation of porous materials at the outlets to absorb the fluid and increase micropumping time [27]. Furthermore, liquid evaporation may also occur after capillary fill and depends on the microchannel dimensions and temperature of the devices [84].

Additionally, durability and stability of microchannels and chemicals also affect the micropumping capability of the capillary flow device, such as reagent degradation over time due to light exposure, moisture or temperature. To circumvent this, the printing of chemicals, freeze-drying chemicals into microchannels, laminations or vacuum sealing can be used [85,86,87].

This article has provided insight for researchers working in the fields of diagnostics to gain an understanding of the capillary-driven flow microfluidics combined with smartphone/portable detections and to familiarize themselves with its potentials in developing point-of-care diagnostic devices. To gain confidence in clinical settings and break through in commercialization requires a reduction in assay steps, no use of sophisticated instruments and less handling expertise, along with highly scalable and inexpensive fabrications. We believe that capillary-driven flow microfluidics holds a true potential in bringing the lab-on-a-chip devices outside the laboratory setting as standalone point-of-care diagnostic platforms.
